# Association of TyG index with prehypertension or hypertension: a retrospective study in Japanese normoglycemia subjects

**DOI:** 10.3389/fendo.2023.1288693

**Published:** 2023-10-27

**Authors:** Jingtao Xu, Weigan Xu, Guojun Chen, Qiaohua Hu, Jun Jiang

**Affiliations:** ^1^ Department of Emergency, First People’s Hospital of Foshan, Foshan, China; ^2^ The Poison Treatment Centre of Foshan, First People’s Hospital of Foshan, Foshan, China

**Keywords:** the triglyceride and glucose (TyG) index, insulin resistance, prehypertension, hypertension, normoglycemia

## Abstract

**Aim:**

The objective of our study was to investigate the potential association between the triglyceride and glucose (TyG) index and the occurrence of prehypertension or hypertension in a cohort of normoglycemic Japanese subjects.

**Methods:**

The NAGALA physical examination program was conducted in 1994 at Murakami Memorial Hospital in Gifu City, Japan. For our retrospective study, we selected 15,450 participants who had taken part in this program. Our aim was to explore the potential link between the TyG index, a surrogate marker for insulin resistance, and the presence of prehypertension (pre-HTN) or hypertension (HTN). Our analysis included adjustments for clinical demographic attributes and serum biomarkers. Logistic regression was employed to assess the relationship between the TyG index and the likelihood of pre-HTN or HTN.

**Results:**

A total of 15,450 study subjects were included in our analysis. Notably, the prevalence of both pre-HTN and HTN displayed an ascending trend with increasing quartiles of the TyG index. In our comprehensive multivariable logistic regression analysis, when evaluating TyG as a continuous variable, the adjusted odds ratio (OR) for pre-HTN was OR 1.31 [95% CI 1.11-1.56], while for HTN, it was OR 1.76 [95% CI 1.24-2.5] within the fully adjusted model (model 3). When TyG was stratified into quartiles within model 3, the adjusted ORs for pre-HTN were OR 1.16 [95% CI 1.02-1.31], OR 1.22 [95% CI 1.06-1.41], and OR 1.31 [95% CI 1.08-1.59], respectively, using quartile 1 as the reference. The adjusted ORs for HTN in quartiles 2, 3, and 4 were OR 1.22 [95% CI 0.89-1.66], OR 1.4 [95% CI 1.02-1.91], and OR 1.48 [95% CI 1.02-2.15], respectively, within the same model and analysis, with quartile 1 as the reference. Subgroup analysis indicated that the TyG index exhibited a significant positive correlation with the risk of hypertension or prehypertension, except in the subgroup aged ≥65 years.

**Conclusion:**

Our study highlights a robust correlation between the TyG index and the likelihood of pre-HTN or HTN in normoglycemic Japanese subjects. This underscores the potential clinical relevance of the TyG index in refining early hypertension management strategies. Nonetheless, the validation of these findings necessitates larger studies with extended follow-up periods.

## 1 Introduction

Hypertension is a disease that seriously endangers human life and health and is widely prevalent worldwide, affecting more than 30% of the population (1). Elevated blood pressure is the leading preventable cause of cardiovascular disease mortality and disease burden globally and in most parts of the world. Hypertension often co-exists with other risk factors and is one of the most important risk factors for ischemic heart disease, stroke, other cardiovascular diseases, chronic kidney disease and dementia (2–6). The etiology of hypertension is complex, and the mechanisms of its cause, occurrence and development are not fully understood. To enhance its prevention and control, scientists are actively searching for risk factors associated with the development of hypertension. Insulin resistance refers to the state of reduced sensitivity and responsiveness of the body to insulin, which has important pathophysiological significance in the development of obesity, T2DM, metabolic syndrome, cardiovascular and cerebrovascular diseases (7). Previous studies have concluded that insulin resistance (IR) is closely associated with hypertension (8). The hyperinsulinemia euglycemic clamp (HIEC) test was first introduced by De Fronzo in 1979 and till date, remains the “gold standard” to assess IR. But the method is more complex and inconvenient to obtain (9). Specifically, the main limitations of the HIEC are that it is time-consuming, labor-intensive, expensive, and requires an experienced operator to manage the technical difficulties, which constrain its usefulness in clinical practice (10). Many simplified mathematical models for assessing insulin resistance have been derived in recent years (11), such as the steady-state model insulin resistance index (HOMA-IR), which is a valid method for assessing insulin resistance calculated from serum glucose and fasting serum insulin (12). However, HOMA-IR requires a serum insulin test, and not all primary care hospitals are able to perform this program, so we need a simpler and more applicable index. Guerrero-Romero et al. (9) proposed and validated a new formula for assessing insulin resistance based on serum triglycerides and fasting glucose levels, namely the triglycerides and glucose (TyG) index. TyG index = ln [fasting serum triglycerides (mg/dL) ×fasting blood glucose (mg/dL)/2], this formula is a potential tool for diagnosing insulin resistance. Previous studies have demonstrated that the TyG index can be an important predictor of the development of metabolic syndrome (13), type 2 diabetes (14), and cardiovascular disease (15, 16). Tao et al. summarized the application value of the TyG index for a variety of CVD types and to explore the potential limitations of using this index as a predictor for cardiovascular diseases (12). But studies on the relationship between TyG and hypertension are less common. In this study, we investigated the relationship between TyG levels and pre-HTN or HTN in Japanese normoglycemic individuals using data from the NAGALA physical examination program (17).

## 2 Methods

### 2.1 Data source

The data used in this work were obtained from the Dryad Digital Repository (www.datadryad.org), which is open to other researchers. Okamura T et al. first explored and made available to the public this original data at https://doi.org/10.5061/dryad. 8q0p192 (17). The variables of database enrolled participants included the following: sex, age, weight, body mass index (BMI), waist circumference (WC), triglycerides (TG), total cholesterol (TC), high-density lipoprotein cholesterol (HDL-C), fasting blood glucose (FPG), hemoglobin A1c (HbA1c), alanine aminotransferase (ALT), aspartate aminotransferase (AST), γ-glutamyl aminotransferase (GGT), systolic blood pressure (SBP), diastolic blood pressure (DBP), smoking status, alcohol consumption, exercise, fatty liver, obesity phenotype, obesity, incident diabetes mellitus (DM), and duration of follow-up. A standardized questionnaire was used to assess the study population’s lifestyle habits. Over the past month, weekly alcohol consumption was classified as none, minimal (<40g/week), light (40-140g/week), moderate (140-280g/week), and high (>280g/week). In terms of smoking status, study participants were classified as current, former, or never smokers. And regular exercise was defined as participating in any type of sport at least once per week.

### 2.2 Study population

In Gifu City, Japan, a healthcare initiative was initiated to identify chronic cardiovascular issues and promote general health. The NAfld in the Gifu Area, Longitudinal Analysis (NAGALA) database was constructed using data collected from this program. The program was performed in 1994 and evaluated over 8000 medical exams annually. Approximately 60% of the participants received one to two exams per year. The study initially encompassed a total of 20,944 participants (12,498 men and 8,446 women). Exclusion criteria included: 1) missing data; 2) fatty liver disease, viral hepatitis B or C at baseline; 3) heavy alcohol consumption (over 60g/d for males and 40g/d for females) at baseline; 4) medication use at baseline; 5) diabetes diagnosis or FPG >6.1 mmol/L at baseline. Murakami Memorial Hospital provided ethical approval for the study, with each patient providing written informed consent for data collection and use. Following the exclusion of cases with abnormal values and those with HDL deficiency, a total of 15,450 cases (8,417 males and 7,033 females) were included in the final analysis.

### 2.3 Measurement of TyG and definition of pre-HTN and HTN

The TyG index was calculated with established formulas according to the previous studies (9): TyG = Ln [TG (mg/dL) *FPG (mg/dL)/2]. According to the Japanese Society of Hypertension Guidelines for the Management of Hypertension (JSH 2019) (18), HTN was defined as SBP ≥140mmHg and/or DBP ≥90mmHg based on the office blood pressure; elevated blood pressure was defined by a SBP or DBP of 120–139 or 80–89 mmHg, respectively; and high normal blood pressure, SBP 120–129 mmHg and DBP <80 mmHg. In our studies, references to previous literature (19), people with elevated blood pressure and high normal blood pressure were classified as pre-HTN.

### 2.4 Statistical analysis

Statistical analyses were carried out using R software (http://www.R-project.org, The R Foundation) and Free Statistics software version 1.3. Normally distributed continuous variables were presented as means ± SD and analyzed using Student’s t-test. Non-normally distributed continuous variables were expressed as median and interquartile range M (Q1–Q3) and evaluated using the Mann-Whitney U-test. Categorical variables were compared using chi-squared tests, with percentages provided. Participants were grouped into TyG quartiles, and statistical differences among groups were assessed using One-way ANOVA for normally distributed data and Kruskal-Wallis tests for non-normally distributed data. Univariate and multivariate logistic regression models were used to estimate the association between baseline TyG index and pre-HTN or HTN, adjusting for confounding variables. Three distinct models were employed: model 1, which was adjusted for sex and age; Model 2, built upon the foundation of model 1, further incorporated variables encompassing WC, smoking status, and alcohol consumption. Model 3 was an extension of model 2, encompassing additional variables of ALT, AST, GGT, TC, and TG levels. Elaborated specifications of these models are meticulously outlined in **Table 1**. All the incorporated covariates were meticulously chosen based on a comprehensive review of previous literature and their clinical significance. To enhance the reliability of outcomes and pinpoint potential interactions, we also carried out pre-defined stratified analyses, categorizing subjects based on sex, age (<65 and ≥65 years), WC (<90 cm in men, <80 cm in women vs. ≥90 cm in men and ≥80 cm in women), BMI (<25 and ≥25 kg/m^2^), alcohol consumption (none, light, moderate, and heavy), and smoking status (never, past, and current). Stratified analyses were also conducted to verify results and identify interactions within specific subgroups. A significance threshold of 0.05 was considered statistically significant.

**Table 1:** Multivariable-adjust ORs and 95%CI of the TyG index quartiles associated with prehypertension or hypertension.

Variable	Unadjusted		Model 1		Model 2		Model 3	
	OR (95%CI)	p value	OR (95%CI)	p value	OR (95%CI)	p value	OR (95%CI)	p value
Prehypertension								
TyG	2.53 (2.38~2.69)	<0.001	1.91 (1.79~2.04)	<0.001	1.43 (1.33~1.53)	<0.001	1.31 (1.11~1.56)	0.002
1st Quartile (≤)	Ref		Ref		Ref		Ref	
2st Quartile (-)	1.78 (1.58~1.99)	<0.001	1.42 (1.27~1.6)	<0.001	1.26 (1.12~1.42)	<0.001	1.16 (1.02~1.31)	0.025
3st Quartile (-)	2.69 (2.4~3)	<0.001	1.85 (1.65~2.08)	<0.001	1.42 (1.26~1.61)	<0.001	1.22 (1.06~1.41)	0.005
4st Quartile (≥)	4.71 (4.22~5.25)	<0.001	2.84 (2.52~3.2)	<0.001	1.76 (1.55~2.01)	<0.001	1.31 (1.08~1.59)	0.007
p for trend	1.66 (1.6~1.71)	<0.001	1.41 (1.36~1.46)	<0.001	1.2 (1.15~1.25)	<0.001	1.09 (1.03~1.16)	0.004
Hypertension								
TyG	4.18 (3.74~4.68)	<0.001	3.04 (2.68~3.44)	<0.001	2.01 (1.76~2.31)	<0.001	1.76 (1.24~2.5)	0.002
1st Quartile (≤)	Ref		Ref		Ref		Ref	
2st Quartile (-)	2.59 (1.95~3.46)	<0.001	1.85 (1.38~2.48)	<0.001	1.45 (1.08~1.97)	0.015	1.22 (0.89~1.66)	0.209
3st Quartile (-)	5.13 (3.91~6.72)	<0.001	2.98 (2.25~3.94)	<0.001	1.91 (1.43~2.56)	<0.001	1.4 (1.02~1.91)	0.037
4st Quartile (≥)	12.22 (9.43~15.84)	<0.001	6 (4.56~7.9)	<0.001	2.86 (2.14~3.82)	<0.001	1.48 (1.02~2.15)	0.041
p for trend	2.25 (2.1~2.41)	<0.001	1.82 (1.68~1.96)	<0.001	1.41 (1.3~1.53)	<0.001	1.13 (1.01~1.27)	0.035

Model 1 adjust for age and sex.

Model 2 adjust for Model 1+WC, Smoking status, Alcohol consumption.

Model 3 adjust for Model 2+ALT, AST, GGT, TC, TG.

Ref, reference; TyG, the triglycerides and glucose index; WC, waist circumference; ALT, alanine aminotransferase; ASL, aspartate aminotransferase; GGT, gamma glutamyl transferase.

## 3 Results

### 3.1 Population

**Figure 1:**
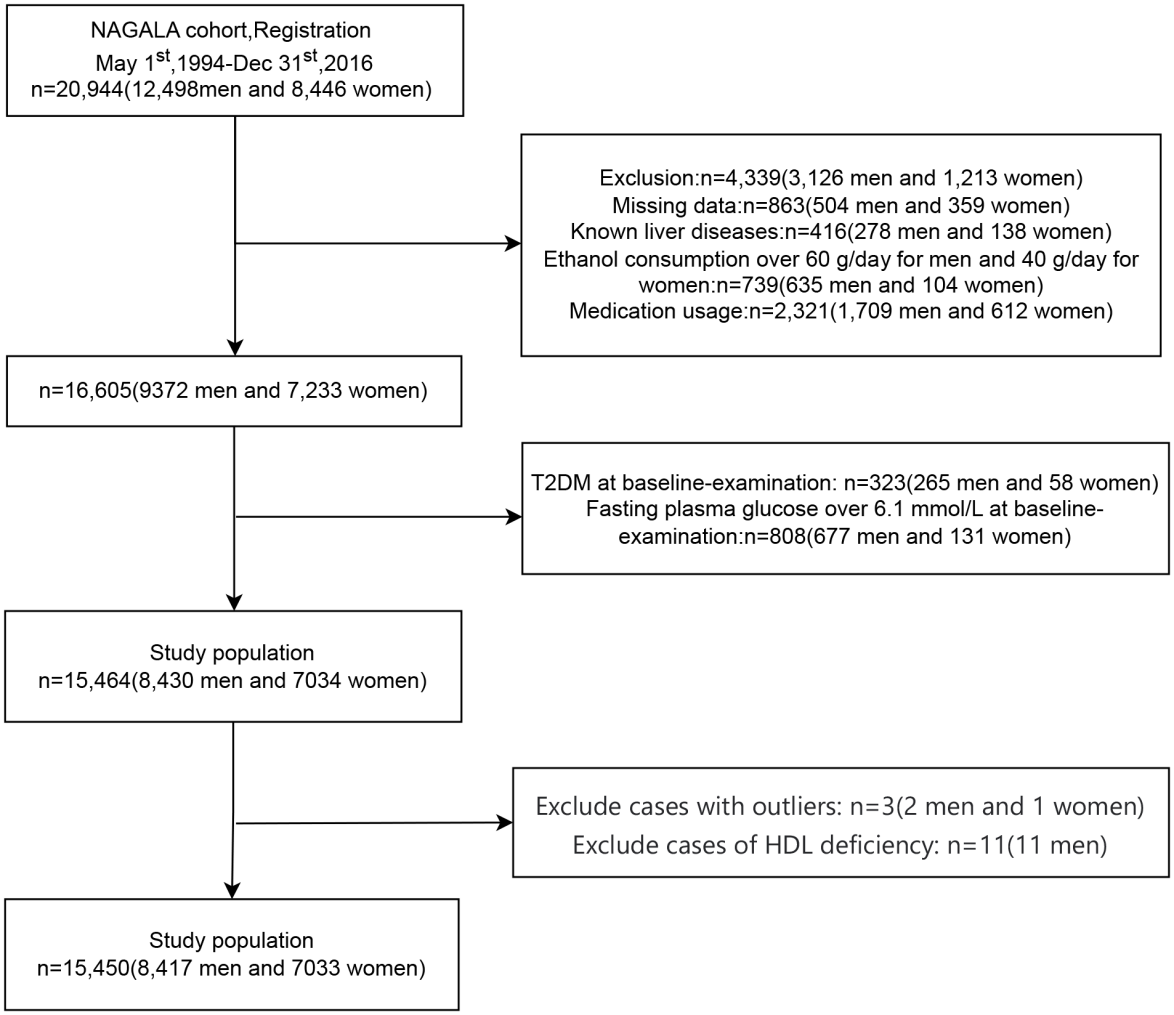
Flowchart of participant selection.

The initial NAGALA cohort encompassed a total of 20,944 individuals. Following the necessary exclusions due to missing data (n=863), known liver disease (n=416), heavy drinking habits (n=739), and baseline medication usage (n=2,321), a total of 4,339 individuals were regrettably excluded. Moreover, during the baseline evaluation, 323 individuals with Type 2 diabetes (T2DM) and 808 individuals with fasting blood glucose levels surpassing 6.1 mmol/L were also deemed ineligible for our study. Subsequently, after the meticulous removal of patients with anomalous values and those exhibiting HDL deficiency, a final cohort of 15,450 cases (8,417 men and 7,033 women) emerged as the foundation for our analytical endeavors. The patient selection flowchart is shown in **Figure 1**.

### 3.2 Baseline characteristics

**Figure 2:**
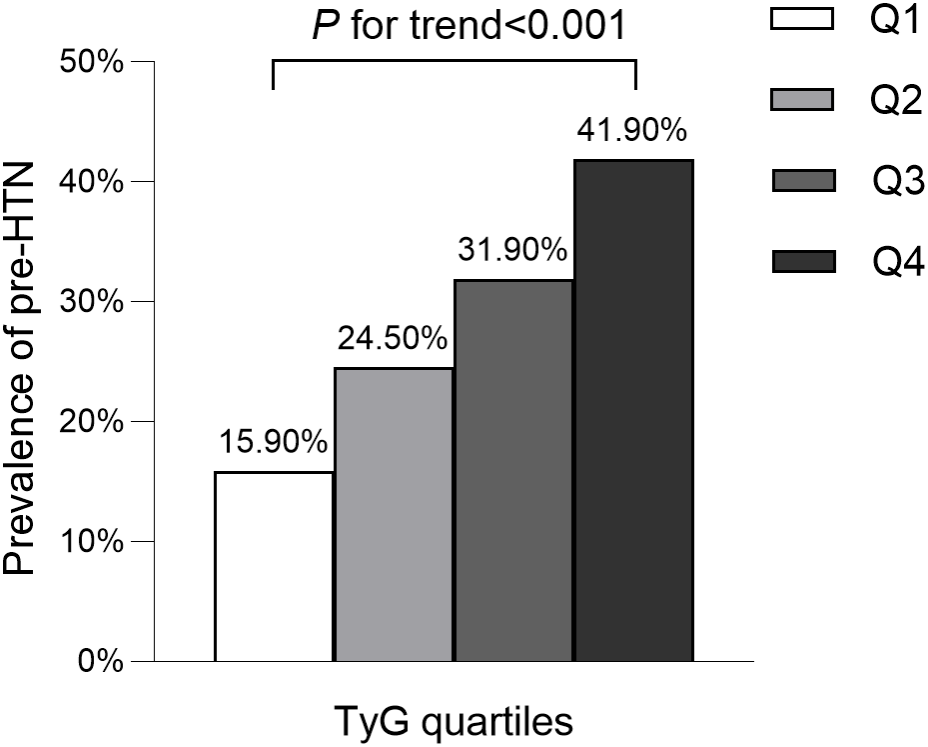
Prevalence of prehypertension according to the baseline TyG quartiles. All of the study participants were divided into four groups according to quartiles of TyG(quartile 1 [Q1]: ≤7.59; quartile 2 [Q2]: 7.60-8.01; quartile 3 [Q3]: 8.02-8.45; quartile 4 [Q4]: ≥8.46). TyG: the triglycerides and glucose index. pre-HTN, prehypertension.

**Figure 3:**
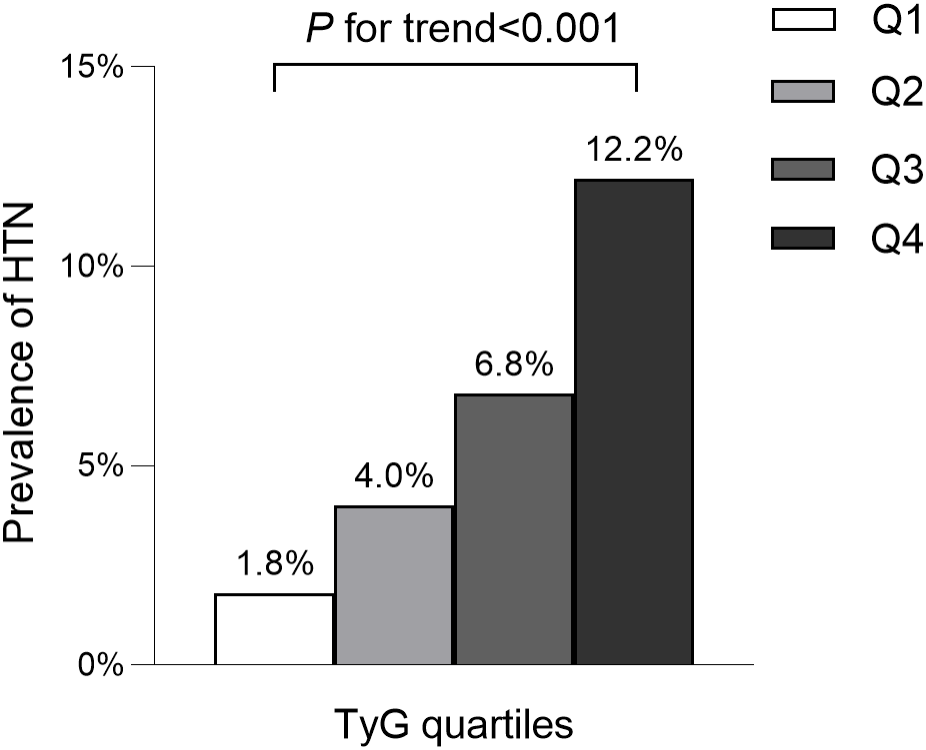
Prevalence of hypertension according to the baseline TyG quartiles. All of the study participants were divided into four groups according to quartiles of TyG(quartile 1 [Q1]: ≤7.59; quartile 2 [Q2]: 7.60-8.01; quartile 3 [Q3]: 8.02-8.45; quartile 4 [Q4]: ≥8.46). TyG: the triglycerides and glucose index. HTN, hypertension.

The average age of participants was 43.7 ± 8.9 years, with 8,417 (54.5%) identified as male. The mean baseline TyG stood at 8.0 ± 0.6. The detailed characteristics of study population stratified by TyG index quartile were shown in **Table 2**. Among the quartiles, individuals in the highest TyG group (Q4) exhibited higher age, body weight, BMI, WC, SBP, and DBP compared to counterparts in the other quartiles (Q1-Q3). Pertaining to laboratory markers, higher TyG levels correlated with elevated TC, TG, ALT, AST, GGT, and FPG levels, while concurrently showing reduced HDL-C levels. Notably, a positive association was identified between higher TyG levels and heavy alcohol consumption as well as smoking. Conversely, a significant inverse correlation was observed between regular exercise and TyG levels. As shown in **Figures 2**, **3**, our analysis delved into the incidence of pre-HTN and HTN within patient groups stratified based on TyG quartiles. Remarkably, an ascending pattern in the prevalence of both pre-HTN and HTN corresponded to increasing TyG quartiles (P for trend 0.001). Specifically, the prevalence of pre-HTN was distributed as follows: 15.9% in Q1, 24.5% in Q2, 31.9% in Q3, and 41.9% in Q4. As for HTN prevalence, the distribution unfolded as 1.8% in Q1, 4.0% in Q2, 6.8% in Q3, and 12.2% in Q4.

**Table 2:** Clinical characteristics of the study population according to TyG.

Variables	Total (n = 15450)	Q1 (n = 3855)	Q2 (n = 3870)	Q3 (n = 3860)	Q4 (n = 3865)	p value
Male, n (%)	8417 (54.5)	961 (24.9)	1816 (46.9)	2459 (63.7)	3181 (82.3)	< 0.001
Age, (years)	43.7 ± 8.9	40.5 ± 8.2	43.5 ± 8.9	45.0 ± 8.9	45.8 ± 8.7	< 0.001
Body weight, (kg)	60.6 ± 11.6	53.7 ± 8.6	58.1 ± 10.0	62.2 ± 10.8	68.5 ± 11.2	< 0.001
BMI, (kg/m^2^)	22.1 ± 3.1	20.4 ± 2.4	21.4 ± 2.7	22.5 ± 3.0	24.1 ± 3.1	< 0.001
WC, (cm)	76.5 ± 9.1	70.8 ± 7.0	74.2 ± 8.0	77.9 ± 8.4	82.9 ± 8.1	< 0.001
Habit of exercise, n (%)						0.002
No	12744 (82.5)	3160 (82)	3131 (80.9)	3202 (83)	3251 (84.1)	
Yes	2706 (17.5)	695 (18)	739 (19.1)	658 (17)	614 (15.9)	
Smoking status, n (%)						< 0.001
Never	9025 (58.4)	2984 (77.4)	2452 (63.4)	2044 (53)	1545 (40)	
Past	2948 (19.1)	466 (12.1)	686 (17.7)	813 (21.1)	983 (25.4)	
Current	3477 (22.5)	405 (10.5)	732 (18.9)	1003 (26)	1337 (34.6)	
Alcohol consumption, n (%)						< 0.001
None	11800 (76.4)	3347 (86.8)	3003 (77.6)	2848 (73.8)	2602 (67.3)	
Light	1754 (11.4)	283 (7.3)	459 (11.9)	500 (13)	512 (13.2)	
Moderate	1357 (8.8)	187 (4.9)	299 (7.7)	367 (9.5)	504 (13)	
Heavy	539 (3.5)	38 (1)	109 (2.8)	145 (3.8)	247 (6.4)	
ALT, (IU/L)	17.0 (13.0, 23.0)	14.0 (11.0, 17.0)	15.0 (12.0, 20.0)	18.0 (14.0, 24.0)	23.0 (17.0, 32.0)	< 0.001
AST, (IU/L)	17.0 (14.0, 21.0)	16.0 (13.0, 19.0)	17.0 (14.0, 20.0)	17.0 (14.0, 21.0)	19.0 (16.0, 24.0)	< 0.001
GGT, (IU/L)	15.0 (11.0, 22.0)	12.0 (10.0, 15.0)	14.0 (11.0, 18.0)	16.0 (12.0, 23.0)	22.0 (16.0, 35.0)	< 0.001
TC, (mg/dL)	198.2 ± 33.4	181.7 ± 29.6	193.6 ± 30.4	202.4 ± 30.8	215.1 ± 33.4	< 0.001
TG, (mg/dL)	65.0 (44.0, 99.0)	34.0 (27.0, 39.0)	54.0 (49.0, 59.0)	79.0 (71.0, 88.0)	136.0 (114.0,173.0)	< 0.001
HDL-c, (mg/dL)	56.5 ± 15.5	65.7 ± 14.7	60.7 ± 14.8	54.3 ± 13.3	45.5 ± 11.2	< 0.001
TG/HDL	1.7 ± 1.7	0.5 ± 0.2	0.9 ± 0.3	1.6 ± 0.5	3.7 ± 2.3	< 0.001
HbA_1_C, (%)	5.2 ± 0.3	5.1 ± 0.3	5.2 ± 0.3	5.2 ± 0.3	5.2 ± 0.3	< 0.001
FPG (mg/dL)	93.0 ± 7.4	88.4 ± 6.7	91.9 ± 6.9	94.4 ± 6.6	97.2 ± 6.6	< 0.001
SBP, (mmHg)	114.5 ± 15.0	107.7 ± 12.8	112.3 ± 14.1	116.3 ± 14.4	121.7 ± 14.9	< 0.001
DBP, (mmHg)	71.6 ± 10.5	66.6 ± 9.1	69.9 ± 9.9	72.9 ± 10.0	76.9 ± 10.3	< 0.001
TyG	8.0 ± 0.6	7.2 ± 0.3	7.8 ± 0.1	8.2 ± 0.1	8.9 ± 0.3	< 0.001

Data were mean ± SD or median (IQR) for skewed variables or numbers (proportions) for categorical variables. BMI, body mass index; WC, waist circumference; ALT, alanine aminotransferase; ASL, aspartate aminotransferase; GGT, gamma glutamyl transferase; TC, total cholesterol; TG, triglyceride; HDL-c, high‐density lipoprotein cholesterol; HbA1c, hemoglobin A1c; FPG, fasting plasma glucose; SBP, systolic blood pressure; DBP, diastolic blood pressure; TyG, the triglycerides and glucose index; Q1, Q2, Q3, and Q4 are quartiles of the triglycerides and glucose index (TyG).

### 3.3 Univariate and multivariate analyses of prehypertension and hypertension

Age, male, weight, BMI, WC, smoking status, alcohol consumption, GGT, TC, TG, HbA1c, FPG and TyG were significantly associated with pre-HTN and HTN (**Table 3**) and were risk factors for the development of pre-HTN and HTN. HDL-C was found to be significantly negatively correlated with pre-HTN and HTN. **Table 3** shows the results of TyG as continuous variables. After adjusting for different confounders, TyG was inversely associated with pre-HTN or HTN in all three models (**Table 1**). The odds ratios (ORs) of TyG were consistently significant in all three models regardless of whether TyG was analyzed as a continuous variable or quartile (OR range 1.16-1.31, p<0.05 for pre-HTN; OR range 1.40-1.76, p<0.05, except quartile 2(Q2) =0.209 for HTN).

**Table 3:** Results of univariate analysis of prehypertension or hypertension.

Variable	Prehypertension		Hypertension	
	OR (95%CI)	p value	OR (95%CI)	p value
Male, n (%)	2.84 (2.63~3.06)	<0.001	3.86 (3.31~4.5)	<0.001
Age, (years)	1.03 (1.03~1.04)	<0.001	1.07 (1.06~1.08)	<0.001
Body weight, (kg)	1.07 (1.07~1.07)	<0.001	1.09 (1.08~1.1)	<0.001
BMI, (kg/m^2^)	1.3 (1.28~1.32)	<0.001	1.44 (1.41~1.47)	<0.001
WC, (cm)	1.1 (1.09~1.1)	<0.001	1.14 (1.13~1.15)	<0.001
Habit of exercise, n (%)				
No	Ref		Ref	
Yes	1 (0.91~1.1)	0.948	1.03 (0.87~1.22)	0.742
Smoking status, n (%)				
Never	Ref		Ref	
Past	1.94 (1.77~2.13)	<0.001	2.08 (1.77~2.44)	<0.001
Current	1.33 (1.22~1.45)	<0.001	1.31 (1.11~1.55)	0.001
Alcohol consumption, n (%)				
None	Ref		Ref	
Light	1.54 (1.38~1.71)	<0.001	1.76 (1.45~2.15)	<0.001
Moderate	1.95 (1.73~2.2)	<0.001	2.91 (2.4~3.54)	<0.001
Heavy	2.57 (2.13~3.1)	<0.001	4.74 (3.63~6.19)	<0.001
HDL-c, (mg/dL)	0.98 (0.98~0.98)	<0.001	0.97 (0.96~0.97)	<0.001
TC, (mg/dL)	1.01 (1.01~1.01)	<0.001	1.01 (1.01~1.02)	<0.001
TG, (mg/dL)	1.01 (1.01~1.01)	<0.001	1.01 (1.01~1.01)	<0.001
HbA1C, (%)	1.71 (1.53~1.92)	<0.001	2.25 (1.83~2.76)	<0.001
FPG (mg/dL)	1.08 (1.08~1.09)	<0.001	1.12 (1.11~1.13)	<0.001
ALT, (IU/L)	1.04 (1.04~1.04)	<0.001	1.04 (1.04~1.05)	<0.001
AST, (IU/L)	1.06 (1.05~1.06)	<0.001	1.06 (1.05~1.07)	<0.001
GGT, (IU/L)	1.03 (1.03~1.03)	<0.001	1.03 (1.03~1.03)	<0.001
TyG	2.53 (2.38~2.69)	<0.001	4.18 (3.74~4.68)	<0.001

BMI, body mass index; WC, waist circumference; HDL-c, high‐density lipoprotein cholesterol; TC, total cholesterol; TG, triglyceride; HbA1c, hemoglobin A1c; FPG, fasting plasma glucose; ALT, alanine aminotransferase; ASL, aspartate aminotransferase; GGT, gamma glutamyl transferase; TyG, the triglycerides and glucose index.

When TyG was evaluated as a continuous variable, the adjusted OR for pre-HTN was OR 1.31 [95% CI 1.11-1.56] and OR 1.76 [95% CI 1.24-2.5] for HTN in the full variables adjusted model (model 3). When TyG was analyzed as quartiles in model 3, the adjusted OR for pre-HTN was OR 1.16 [95% CI 1.02-1.31], OR 1.22 [95% CI 1.06-1.41], and OR 1.31 [95% CI 1.08-1.59], respectively, with quartile 1 as reference. The adjusted ORs for HTN in Q2, 3, and 4 were OR 1.22 [95% CI 0.89-1.66], OR 1.4 [95% CI 1.02-1.91], and OR 1.48 [95% CI 1.02-2.15], respectively, in the same model and analysis, with quartile 1 as the reference. Furthermore, it was statistically significant in all models (**Table 1**, p for trend<0.001), indicating that TyG was positively associated with pre-HTN and HTN.

### 3.4 Subgroup analyses by adjusted potential effect confounders

**Figure 4:**
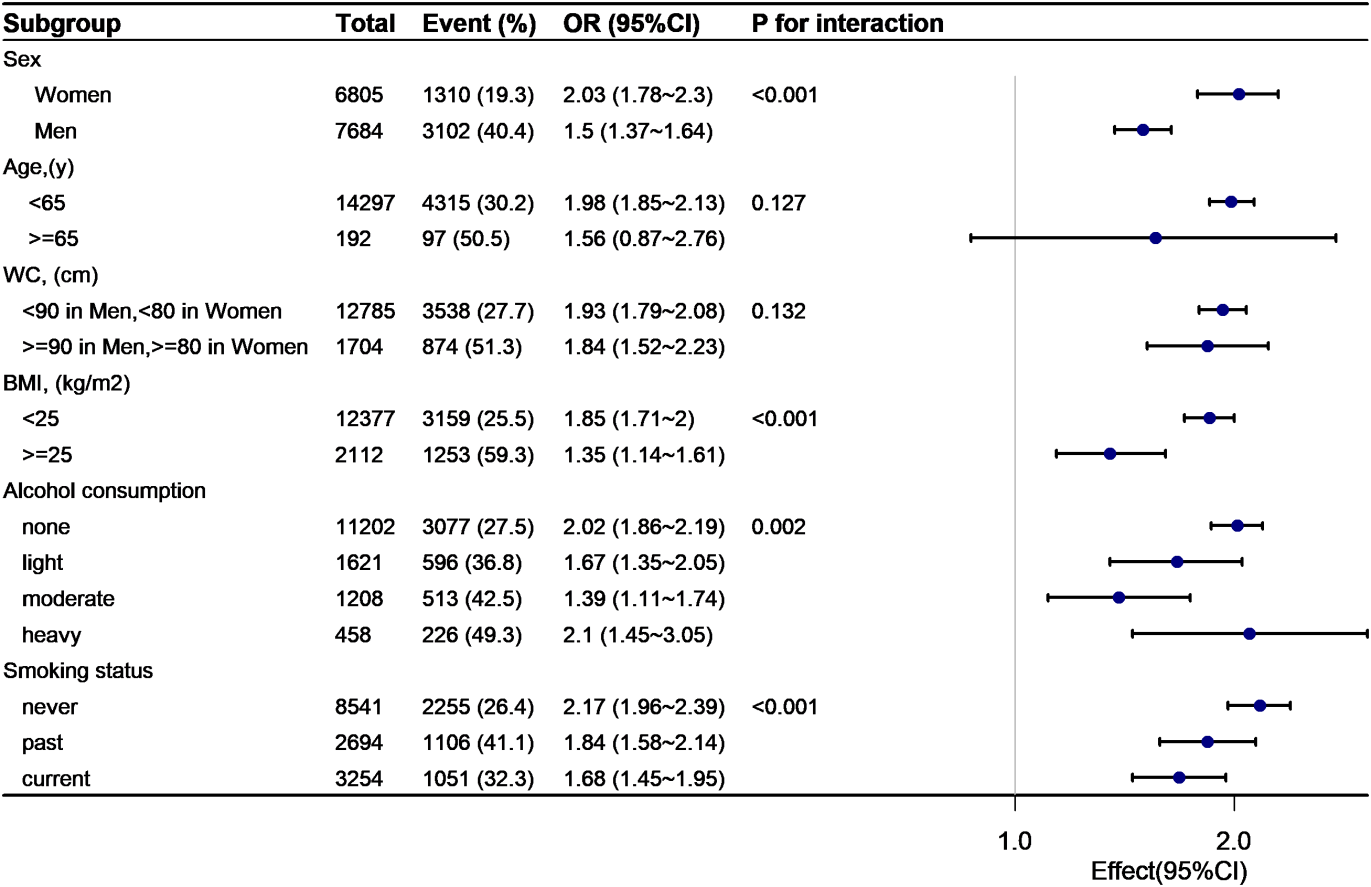
Subgroup analysis of the TyG and prehypertension.

**Figure 5:**
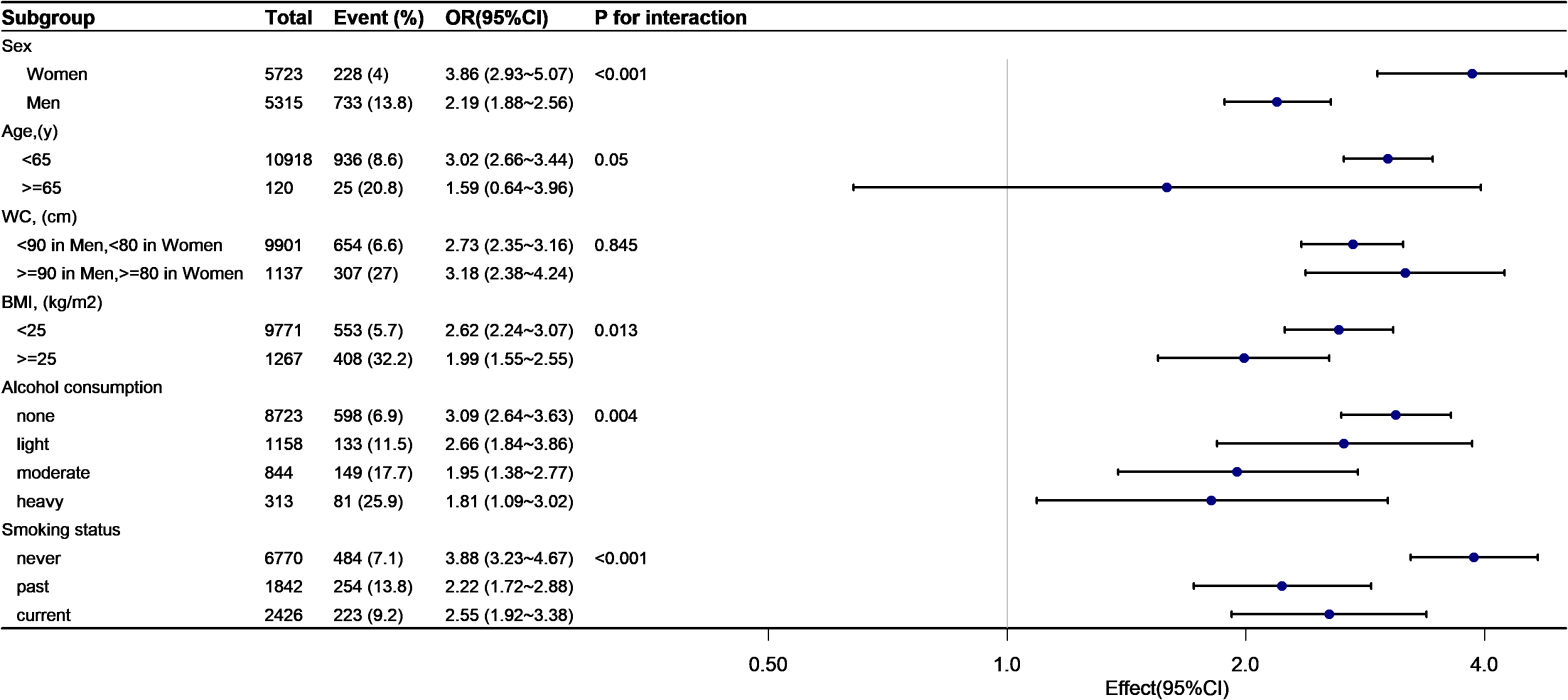
Subgroup analysis of the TyG and hypertension.

As shown in **Figures 4**, **5**, subgroup analyses were carried out to determine the effect of TyG (per 1-unit increment) on pre-HTN and HTN in different subgroups. The relationship between TyG and pre-HTN or HTN was coordinated in the following subgroups: in pre-HTN, sex(female vs. male; P-interaction < 0.001), age (<65 years vs. ≥65 years; P-interaction = 0.127), WC (<90 cm in men, <80 cm in women vs. ≥90 cm in men and ≥80 cm in women; P-interaction = 0.132), and BMI (<24 kg/m^2^ vs. ≥24 kg/m^2^; P-interaction < 0.001); in HTN, sex(female vs. male; P-interaction < 0.001), age (<65 years vs. ≥65 years; P-interaction = 0.05), WC (<90 cm in men, <80 cm in women vs. ≥90 cm in men and ≥80 cm in women; P-interaction = 0.845), and BMI (<24 kg/m^2^ vs. ≥24 kg/m^2^; P-interaction = 0.013).

## 4 Discussion

In this cross-sectional study based on a Japanese normoglycemic population, we explored the association of TyG, a surrogate indicator of non-insulin-based IR, with hypertension and prehypertension. Our results show that the prevalence of HTN and pre-HTN increased progressively with increasing TyG quartile index. Meanwhile, this study also found that the change value of TyG also had a nonlinear response relationship with the risk of hypertension. The incidence of hypertension was increased by 122% in the Q2 group compared with the Q1 group. The incidence of hypertension was increased by 70% in the Q3 group compared with the Q2 group. The incidence of hypertension was increased by 79.4% in the Q4 group compared with the Q3 group. Among this four groups, the incidence of hypertension increased at different rates. The mechanism of these results need to be further investigated. Further analysis of the study revealed that TyG index was positively associated with the risk of developing hypertension and pre-HTN both as a continuous variable and as a categorical variable. The results remained the same after subgroup analysis by age, sex, WC and BMI.

Insulin resistance is defined as decreased responsiveness (maximal insulin effect) or sensitivity (insulin concentration required for a half maximal response) to insulin’s metabolic actions (20).

Studies in epidemiology and basic science have showed that IR is one of the potential factor of prehypertension (21, 22) and some researches also suggested that IR is one of the significant risk factor of hypertension (23, 24). However, the detection of IR is complex and difficult to obtain (9). Hence, some recent studies (25, 26) have suggested that triglyceride glucose product index (TyG) can be an alternative index of IR. The results of cross-sectional studies have shown that TyG has the highest sensitivity and specificity. Compared with some past indexes of IR, such as steady-state model insulin resistance index (HOMA-IR) and TG/HDL-C, TyG has the highest sensitivity and specificity for the diagnosis of IR. The sensitivity for the diagnosis of IR is 84%. The specificity for the diagnosis of IR is 45%. These results showed that the diagnostic accuracy of TyG is better (27). Previous research on the relationship between TyG and prehypertension has been limited. Rongjiong Zheng et al. (23) studied 4686 subjects and followed up for 9 years showed that The TyG index was found to be a good predictor of incident hypertension, and Cox regression analyses revealed that a higher TyG index was linked to an increased risk of incident hypertension. A study (28) that included 32,124 normoglycemic adults showed that when comparing the highest TyG index to the lowest TyG index, there was an association with prehypertension and hypertension, with corresponding ORs of 1.795 (1.638, 1.968) and 2.439 (2.205,2.698), respectively.

Xin Zhang et al. (29) also reported that noninsulin-based IR indexes (TG/HDL-C, TyG, and METS-IR) are significantly related with the risk of prehypertension. However, Jie Fan, MB et al. (21) discovered that METS-IR, but not TG/HDL or TyG, was significantly associated with prehypertension, with a 2.223 odds ratio for prehypertension in the highest quartile versus the lowest. In this study, we found that with increasing TyG quartiles, the prevalence of both pre-HTN and HTN increased. The logistic regression analysis showed that age, male, weight, BMI, WC, smoking status, alcohol consumption, GGT, TC, TG, HbA1c, FPG, and TyG were all significantly associated with pre-HTN and HTN (**Table 3**) and were risk factors for pre-HTN and HTN development. After adjusting for different confounders, TyG was positively related to pre-HTN and HTN in all three models, regardless of whether TyG was analyzed as a continuous variable or in quartiles.

There are several possible mechanisms by which excessive TyG may increase the risk of hypertension. To begin, IR causes a decrease in the efficiency of glucose uptake and utilization by insulin in the body, leading to compensatory overproduction of insulin by the body, which increases sympathetic nervous system activity and promotes the body to secrete more epinephrine and norepinephrine, ultimately increasing cardiac output and peripheral vascular resistance (30, 31). Secondly, IR may also increase the synthesis and release of endothelin, which may contract blood vessels, and decrease the synthesis of prostacyclin (PGI2) and prostaglandin E2 (PGE2), which may dilate vessels (32, 33), as well as induce the proliferation of vascular smooth muscle, which may result in an increase in blood pressure. Thirdly, when insulin resistance causes hyperinsulinemia, sodium reabsorption from the renal tubules increases, resulting in high blood pressure (34, 35). Besides, as two components of TyG, both TG and FPG are closely associated with the development of hypertension (36, 37). It has been shown that dyslipidemia is observed in 50%-80% of hypertensive patients (38).

Nevertheless, several limitations also exist in this study, which should be acknowledged. First and foremost, because the current study was cross-sectional in nature, causal correlations between TyG and hypertension and prehypertension cannot be drawn. Second, this study was limited to the Japanese population. As a result, to evaluate generalizability to other races and people with varied origins, our findings need be replicated in other cohorts. Third, TyG were discovered in Caucasian and Mexican cultures, and the insulin secretory capacity of East Asians differs from that of other ethnicities (29). Furthermore, because the secondary analysis was constrained by the available data, unreported or unmeasured confounding factors in the original study could not be adequately adjusted. It is well known that uric acid and renal function have a synergistic effect on insulin resistance and vascular damage in hypertensive patients (39). In addition, inflammation is a key factor in worsening insulin resistance and arterial hypertension (40). These variables may have a potential impact on the results of the study. Given these limitations, further prospective cohort studies should be carried out to clarify the above factors. However, the large sample size of this study ensures that the results are relatively robust and reliable.

In summary, our study showed that the insulin resistance index TyG was strongly associated with the risk of pre-HTN or HTN in normoglycemic Japanese subjects, suggesting that TyG is clinically important for the early refinement of hypertension management. Besides, our findings raise the possibility that maybe we can reduce hypertension by controlling FPG and TG to lower the TyG index. However, larger studies with longer follow-up are required to confirm.

## Data availability statement

The datasets presented in this study can be found in online repositories. The names of the repository/repositories and accession number(s) can be found in the article/supplementary material.

## Author contributions

JX: Writing – original draft. WX: Writing – review & editing. GC: Writing – review & editing. QH: Writing – review & editing. JJ: Writing – review & editing.

## References

[1] Mills KT Stefanescu A He J. The global epidemiology of hypertension. Nat Rev Nephrol (2020) 16(4):223–37. doi: 10.1038/s41581-019-0244-2 PMC7998524 32024986

[2] Zhou B Perel P Mensah GA Ezzati M. Global epidemiology, health burden and effective interventions for elevated blood pressure and hypertension. Nat Rev Cardiol (2021) 18(11):785–802. doi: 10.1038/s41569-021-00559-8 34050340 PMC8162166

[3] Agbor-Etang BB Setaro JF. Management of hypertension in patients with ischemic heart disease. Curr Cardiol Rep (2015) 17(12):119. doi: 10.1007/s11886-015-0662-0 26482762

[4] Buonacera A Stancanelli B Malatino L. Stroke and hypertension: an appraisal from pathophysiology to clinical practice. Curr Vasc Pharmacol (2019) 17(1):72–84. doi: 10.2174/1570161115666171116151051 29149815

[5] Gargiulo R Suhail F Lerma EV. Hypertension and chronic kidney disease. Disease-a-month DM (2015) 61(9):387–95. doi: 10.1016/j.disamonth.2015.07.003 26328515

[6] Walker KA Power MC Gottesman RF. Defining the relationship between hypertension, cognitive decline, and dementia: a review. Curr Hypertension Rep (2017) 19(3):24. doi: 10.1007/s11906-017-0724-3 PMC6164165 28299725

[7] Adeva-Andany MM Martínez-Rodríguez J González-Lucán M Fernández-Fernández C Castro-Quintela E. Insulin resistance is a cardiovascular risk factor in humans. Diabetes Metab Syndrome (2019) 13(2):1449–55. doi: 10.1016/j.dsx.2019.02.023 31336505

[8] Wang F Han L Hu D. Fasting insulin, insulin resistance and risk of hypertension in the general population: A meta-analysis. Clin Chim Acta Int J Clin Chem (2017) 464:57–63. doi: 10.1016/j.cca.2016.11.009 27836689

[9] Guerrero-Romero F Simental-Mendía LE González-Ortiz M Martínez-Abundis E Ramos-Zavala MG Hernández-González SO. The product of triglycerides and glucose, a simple measure of insulin sensitivity. Comparison with the euglycemic-hyperinsulinemic clamp. J Clin Endocrinol Metab (2010) 95(7):3347–51. doi: 10.1210/jc.2010-0288 20484475

[10] Minh HV Tien HA Sinh CT Thang DC Chen CH Tay JC. Assessment of preferred methods to measure insulin resistance in Asian patients with hypertension. J Clin Hypertension (Greenwich Conn) (2021) 23(3):529–37. doi: 10.1111/jch.14155 PMC8029536 33415834

[11] Gutch M Kumar S Razi SM Gupta KK Gupta A. Assessment of insulin sensitivity/resistance. Indian J Endocrinol Metab (2015) 19(1):160–4. doi: 10.4103/2230-8210.146874 PMC4287763 25593845

[12] Janchevska A Gucev Z Tasic V Polenakovic M. Homeostasis model assessment - insulin resistance and sensitivity (HOMA-IR and IS) index in overweight children born small for gestational age (SGA). Prilozi (Makedonska Akademija Na Naukite I Umetnostite Oddelenie Za Medicinski Nauki) (2018) 39(1):83–9. doi: 10.2478/prilozi-2018-0027 30110267

[13] Aslan Çin NN Yardımcı H Koç N Uçaktürk SA Akçil Ok M. Triglycerides/high-density lipoprotein cholesterol is a predictor similar to the triglyceride-glucose index for the diagnosis of metabolic syndrome using International Diabetes Federation criteria of insulin resistance in obese adolescents: a cross-sectional study. J Pediatr Endocrinol Metab JPEM (2020) 33(6):777–84. doi: 10.1515/jpem-2019-0310 32447329

[14] Su WY Chen SC Huang YT Huang JC Wu PY Hsu WH. Comparison of the effects of fasting glucose, hemoglobin A(1c), and triglyceride-glucose index on cardiovascular events in type 2 diabetes mellitus. Nutrients (2019) 11(11):2838. doi: 10.3390/nu11112838 31752391 PMC6893677

[15] Park K Ahn CW Lee SB Kang S Nam JS Lee BK. Elevated tyG index predicts progression of coronary artery calcification. Diabetes Care (2019) 42(8):1569–73. doi: 10.2337/dc18-1920 31182490

[16] Tao LC Xu JN Wang TT Hua F Li JJ. Triglyceride-glucose index as a marker in cardiovascular diseases: landscape and limitations. Cardiovasc Diabetol (2022) 21(1):68. doi: 10.1186/s12933-022-01511-x 35524263 PMC9078015

[17] Okamura T Hashimoto Y Hamaguchi M Obora A Kojima T Fukui M. Ectopic fat obesity presents the greatest risk for incident type 2 diabetes: a population-based longitudinal study. Int J Obes (Lond) (2019) 43(1):139–48. doi: 10.1038/s41366-018-0076-3 29717276

[18] Umemura S Arima H Arima S Asayama K Dohi Y Hirooka Y. The Japanese society of hypertension guidelines for the management of hypertension (JSH 2019). Hypertension Res (2019) 42(9):1235–481. doi: 10.1038/s41440-019-0284-9 31375757

[19] Han KY Gu J Wang Z Liu J Zou S Yang CX. Association between METS-IR and prehypertension or hypertension among normoglycemia subjects in Japan: A retrospective study. Front Endocrinol (2022) 13:851338. doi: 10.3389/fendo.2022.851338 PMC8971288 35370984

[20] Park SY Gautier JF Chon S. Assessment of insulin secretion and insulin resistance in human. Diabetes Metab J (2021) 45(5):641–54. doi: 10.4093/dmj.2021.0220 PMC8497920 34610719

[21] Fan J Gao ST Wang LJ Qian ZL Zhou ZQ Liu XZ. Association of three simple insulin resistance indexes with prehypertension in normoglycemic subjects. Metab Syndrome Related Disord (2019) 17(7):374–9. doi: 10.1089/met.2019.0029 31211636

[22] Li R Zhang H Wang W Wang X Huang Y Huang C. Vascular insulin resistance in prehypertensive rats: role of PI3-kinase/Akt/eNOS signaling. Eur J Pharmacol (2010) 628(1-3):140–7. doi: 10.1016/j.ejphar.2009.11.038 19944677

[23] Zheng R Mao Y. Triglyceride and glucose (TyG) index as a predictor of incident hypertension: a 9-year longitudinal population-based study. Lipids Health Dis (2017) 16(1):175. doi: 10.1186/s12944-017-0562-y 28903774 PMC5598027

[24] Mancusi C Izzo R di Gioia G Losi MA Barbato E Morisco C. Insulin resistance the hinge between hypertension and type 2 diabetes. High Blood Press Cardiovasc Prev (2020) 27(6):515–26. doi: 10.1007/s40292-020-00408-8 PMC7661395 32964344

[25] Ramdas Nayak VK Satheesh P Shenoy MT Kalra S. Triglyceride Glucose (TyG) Index: A surrogate biomarker of insulin resistance. JPMA J Pakistan Med Assoc (2022) 72(5):986–8. doi: 10.47391/JPMA.22-63 35713073

[26] Liao Y Zhang R Shi S Zhao Y He Y Liao L. Triglyceride-glucose index linked to all-cause mortality in critically ill patients: a cohort of 3026 patients. Cardiovasc Diabetol (2022) 21(1):128. doi: 10.1186/s12933-022-01563-z 35804386 PMC9270811

[27] Simental-Mendía LE Rodríguez-Morán M Guerrero-Romero F. The product of fasting glucose and triglycerides as surrogate for identifying insulin resistance in apparently healthy subjects. Metab Syndrome Related Disord (2008) 6(4):299–304. doi: 10.1089/met.2008.0034 19067533

[28] Zhang F Zhang Y Guo Z Yang H Ren M Xing X. The association of triglyceride and glucose index, and triglyceride to high-density lipoprotein cholesterol ratio with prehypertension and hypertension in normoglycemic subjects: A large cross-sectional population study. J Clin Hypertension (Greenwich Conn) (2021) 23(7):1405–12. doi: 10.1111/jch.14305 PMC8678664 34118112

[29] Zhang X Yu C Ye R Liu T Chen X. Correlation between non-insulin-based insulin resistance indexes and the risk of prehypertension: A cross-sectional study. J Clin Hypertension (Greenwich Conn) (2022) 24(5):573–81. doi: 10.1111/jch.14449 PMC9106071 35411676

[30] Tack CJ Smits P Willemsen JJ Lenders JW Thien T Lutterman JA. Effects of insulin on vascular tone and sympathetic nervous system in NIDDM. Diabetes (1996) 45(1):15–22. doi: 10.2337/diab.45.1.15 8522054

[31] da Silva AA do Carmo JM Li X Wang Z Mouton AJ Hall JE. Role of hyperinsulinemia and insulin resistance in hypertension: metabolic syndrome revisited. Can J Cardiol (2020) 36(5):671–82. doi: 10.1016/j.cjca.2020.02.066 PMC7219403 32389340

[32] Konukoglu D Uzun H. Endothelial dysfunction and hypertension. Adv Exp Med Biol (2017) 956:511–40. doi: 10.1007/5584_2016_90 28035582

[33] Axelrod L. Insulin, prostaglandins, and the pathogenesis of hypertension. Diabetes (1991) 40(10):1223–7. doi: 10.2337/diab.40.10.1223 1936584

[34] Rett K Wicklmayr M Mehnert H. New aspects of insulin resistance in hypertension. Eur Heart J (1994) 15(Suppl C):78–81. doi: 10.1093/eurheartj/15.suppl_C.78 7995275

[35] Ohishi M. Hypertension with diabetes mellitus: physiology and pathology. Hypertension Res (2018) 41(6):389–93. doi: 10.1038/s41440-018-0034-4 29556093

[36] He D Fan F Jia J Jiang Y Sun P Wu Z. Lipid profiles and the risk of new-onset hypertension in a Chinese community-based cohort. Nutrition Metabol Cardiovasc Dis NMCD (2021) 31(3):911–20. doi: 10.1016/j.numecd.2020.11.026 33549431

[37] Geva M Shlomai G Berkovich A Maor E Leibowitz A Tenenbaum A. The association between fasting plasma glucose and glycated hemoglobin in the prediabetes range and future development of hypertension. Cardiovasc Diabetol (2019) 18(1):53. doi: 10.1186/s12933-019-0859-4 31029146 PMC6486972

[38] O'Meara JG Kardia SL Armon JJ Brown CA Boerwinkle E Turner ST. Ethnic and sex differences in the prevalence, treatment, and control of dyslipidemia among hypertensive adults in the GENOA study. Arch Internal Med (2004) 164(12):1313–8. doi: 10.1001/archinte.164.12.1313 15226165

[39] Cassano V Crescibene D Hribal ML Pelaia C Armentaro G Magurno M. Uric acid and vascular damage in essential hypertension: role of insulin resistance. Nutrients (2020) 12(9):2509. doi: 10.3390/nu12092509 32825165 PMC7551393

[40] Cassano V Tripepi G Perticone M Miceli S Scopacasa I Armentaro G. Endothelial progenitor cells predict vascular damage progression in naive hypertensive patients according to sex. Hypertension Res (2021) 44(11):1451–61. doi: 10.1038/s41440-021-00716-z 34471254

